# Ocean Warming Enhances Malformations, Premature Hatching, Metabolic Suppression and Oxidative Stress in the Early Life Stages of a Keystone Squid

**DOI:** 10.1371/journal.pone.0038282

**Published:** 2012-06-06

**Authors:** Rui Rosa, Marta S. Pimentel, Joana Boavida-Portugal, Tatiana Teixeira, Katja Trübenbach, Mário Diniz

**Affiliations:** 1 Laboratório Marítimo da Guia, Centro de Oceanografia, Faculdade de Ciências da Universidade de Lisboa, Cascais, Portugal; 2 REQUIMTE, Departamento de Química, Centro de Química Fina e Biotecnologia, Faculdade de Ciências e Tecnologia, Universidade Nova de Lisboa, Caparica, Portugal; Institute of Marine Research, Norway

## Abstract

**Background:**

The knowledge about the capacity of organisms’ early life stages to adapt to elevated temperatures is very limited but crucial to understand how marine biota will respond to global warming. Here we provide a comprehensive and integrated view of biological responses to future warming during the early ontogeny of a keystone invertebrate, the squid *Loligo vulgaris*.

**Methodology/Principal Findings:**

Recently-spawned egg masses were collected and reared until hatching at present day and projected near future (+2°C) temperatures, to investigate the ability of early stages to undergo thermal acclimation, namely phenotypic altering of morphological, behavioural, biochemical and physiological features. Our findings showed that under the projected near-future warming, the abiotic conditions inside the eggs promoted metabolic suppression, which was followed by premature hatching. Concomitantly, the less developed newborns showed greater incidence of malformations. After hatching, the metabolic burst associated with the transition from an encapsulated embryo to a planktonic stage increased linearly with temperature. However, the greater exposure to environmental stress by the hatchlings seemed to be compensated by physiological mechanisms that reduce the negative effects on fitness. Heat shock proteins (HSP70/HSC70) and antioxidant enzymes activities constituted an integrated stress response to ocean warming in hatchlings (but not in embryos).

**Conclusions/Significance:**

The stressful abiotic conditions inside eggs are expected to be aggravated under the projected near-future ocean warming, with deleterious effects on embryo survival and growth. Greater feeding challenges and the lower thermal tolerance limits of the hatchlings are strictly connected to high metabolic demands associated with the planktonic life strategy. Yet, we found some evidence that, in the future, the early stages might support higher energy demands by adjusting some cellular functional properties to increase their thermal tolerance windows.

## Introduction

The Earth’s climate has warmed by approximately 0.6°C over the past century, and the net heat uptake by the world’s ocean has been more than 20 times greater than that by the atmosphere [1]. Average global sea surface temperatures are expected to increase up to 3°C by 2100 [2], which is predicted to drive profound impacts on marine phenology [3], species richness [4], physiology [5]–[8] and biogeography [9].

Coastal marine ecosystems are among the most socio-economically and ecologically important habitats, and they are warming at a much faster rate than many other ecosystems [10]. Because many coastal organisms already live close to their thermal tolerance limits [11], [12], ocean warming will negatively impact their performance and survival. It is known that the metabolism of marine ectotherms is constrained by oxygen supply at high (and low) temperatures with a progressive transition to an anaerobic mode of energy production (the “oxygen limitation of thermal tolerance” concept [5], [13]). The reduction in aerobic scope is not caused by lower levels of ambient oxygen but through limited capacity of oxygen supply mechanisms (ventilatory and circulatory systems) to meet an animal’s temperature-dependent oxygen demand. In this context, scientists are increasingly being called upon to predict how physiological acclimation to increased future temperatures may affect behavior, growth and reproduction and possibly shape the long-term fate of species. For example, the aerobic scope of some tropical reef fishes is known to decrease when water temperature is raised above normal summer temperature, limiting their development and reproductive capacity [8], [14], [15]. Yet, most research has been conducted on adult stages, although early stages are expected to be the most vulnerable [16]–[19] and may constitute a bottleneck for species survival (see review in [20]).

Squids play an important role as prey and predators in marine ecosystems [6], [21], and are usually defined as keystone species because, despite being less abundant than some other invertebrate species, they exert a strong influence on the ecosystem dynamics. Some squid species also function as important biological pumps, conveying substantial biomass between pelagic and coastal ecosystems [22]. As in other marine species, squid’s early life history is characterized by high mortality and strong selection pressures. Yet, the early stages are not only influenced by the external environment but also by the intrinsic embryo characteristics inherited from their parents [19]. In the case of the European squid, *Loligo vulgaris*, its extended spawning season (with two peaks, a major one in February and a minor one in June) and the marked environmental seasonality in western Iberian waters [23] implies that squid hatched in distinct periods experience extremely different thermal conditions. Moreover, it is known that the winter spawners lay smaller eggs than the summer ones, in which the advantage may lie in a higher number of offspring surviving natural mortality, rather than higher individual fitness [24].

But, what is the ability of *L. vulgaris’* early stages to undergo thermal acclimation (involving phenotypic altering of morphological, behavioural, biochemical and physiological characteristics) as a means of coping with the predicted warming? Temperature is known to be the main factor regulating the duration of squid embryonic development [25], [26] and a source of paralarval morphological variation [27], [28]. With elevated temperature, heat shock protein (HSP) production may be induced several hundred fold to repair, refold, and eliminate damaged or denatured proteins mechanisms [29]. Additionally, the expected increase in metabolic demands with concomitant ROS formation may be followed by an enhancement in the activity of a powerful repertoire of antioxidant enzymes, including superoxide dismutase (SOD), which converts O_2_^−^ in H_2_O_2_, catalase (CAT) which remove H_2_O_2_ avoiding its accumulation in cells and tissues and glutathione-S-transferase GST that transform xenobiotics into other conjugates [30]. Within this context, here we provide a comprehensive and integrated view of biological responses to global warming (under a realistic scenario), during squid’s early ontogeny. Recently-spawned *L. vulgaris* egg masses were collected in cooler (winter) and warmer (summer) periods, and reared until hatching at present day (between 13°C and 17°C) and future (+2°C, [31]) temperatures in the western Iberian coast in 2100. We examined how the different thermal scenarios affect: i) survival rates and development time, ii) embryo growth and yolk-sac depletion, iii) hatching (incidence of malformations and premature paralarvae), iv) metabolic rates and thermal sensitivity (Q_10_ values), v) anaerobic pathways (octopine concentration), vi) thermal tolerance limits (LT50 and LT100), vii) heat shock response (HSP70/HSC70), and viii) oxidative stress (activity of several antioxidative enzymes) and “peroxidation” (malondialdehyde concentration).

## Results

### Survival and Development Time

**Figure 1:**
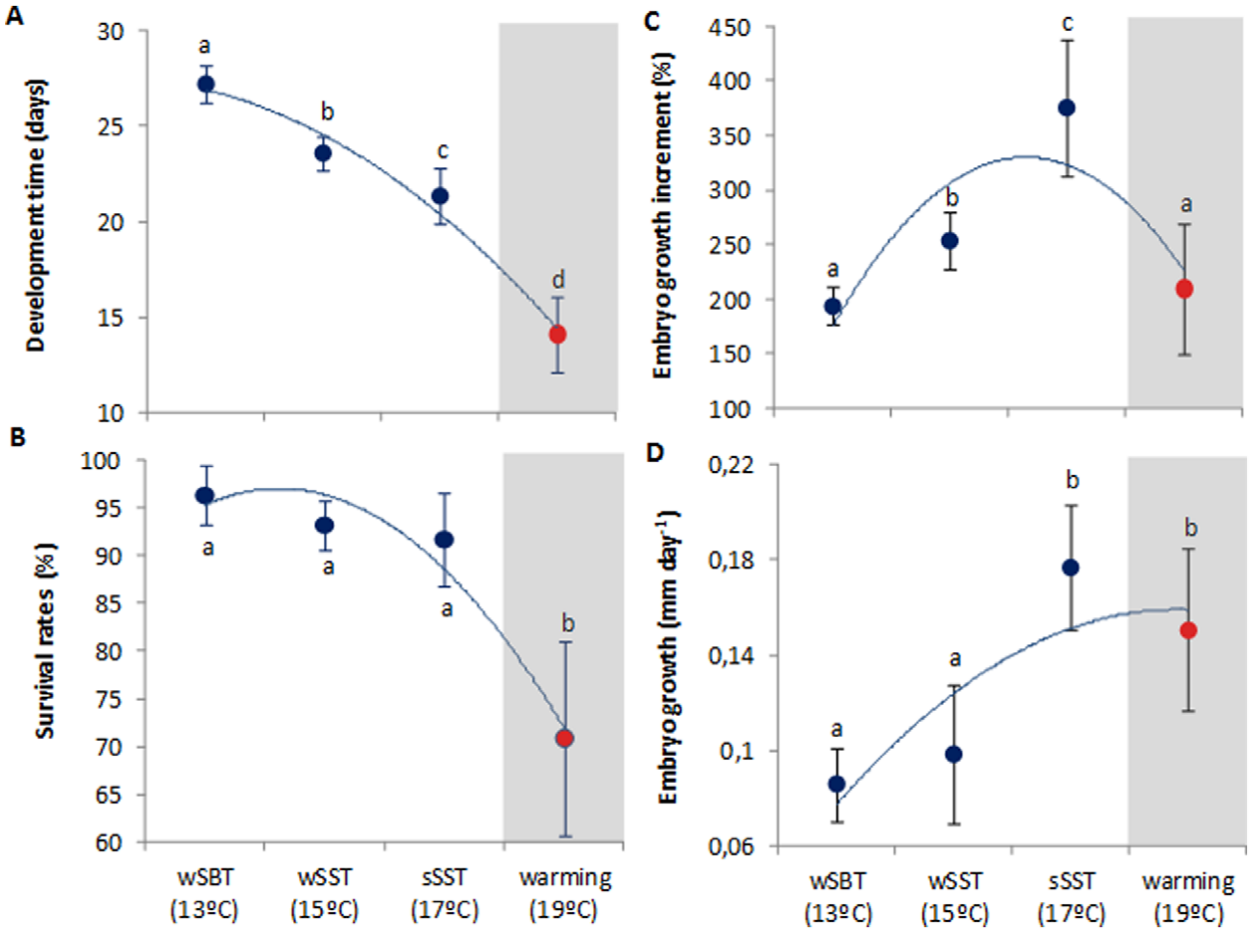
Effect of warming in the early ontogeny squid, Loligo vulgaris, namely: Development time (A), survival rates (B) and growth rates (% - C; mm day−1 - D) of embryos at the different temperature scenarios (red symbols highlight the future summer scenario). Values are mean ± SD. Blue lines represent trendlines and different letters represent significant differences (more statistical details in Supporting Tables).

As expected, the duration of the embryonic period in *L. vulgaris* was significantly shortened with increasing temperature (Fig. 1A, F = 459.0, p<0.001). During the winter season, embryogenesis lasted 27±1 days; yet, the summer embryos incubated in the future warming scenario (19°C) hatched after 14±2 days. The survival rates ranged between 96% and 92% under the present-day scenarios (between 13°C and 17°C, respectively). However, the projected near-future ocean warming elicited a significant negative effect, with survival rates decreasing up to 71% (red symbol, Fig. 1B, F = 4.22, p = 0.046, see also Table S1).

### Growth, Abnormalities and Premature Hatching

**Figure 2:**
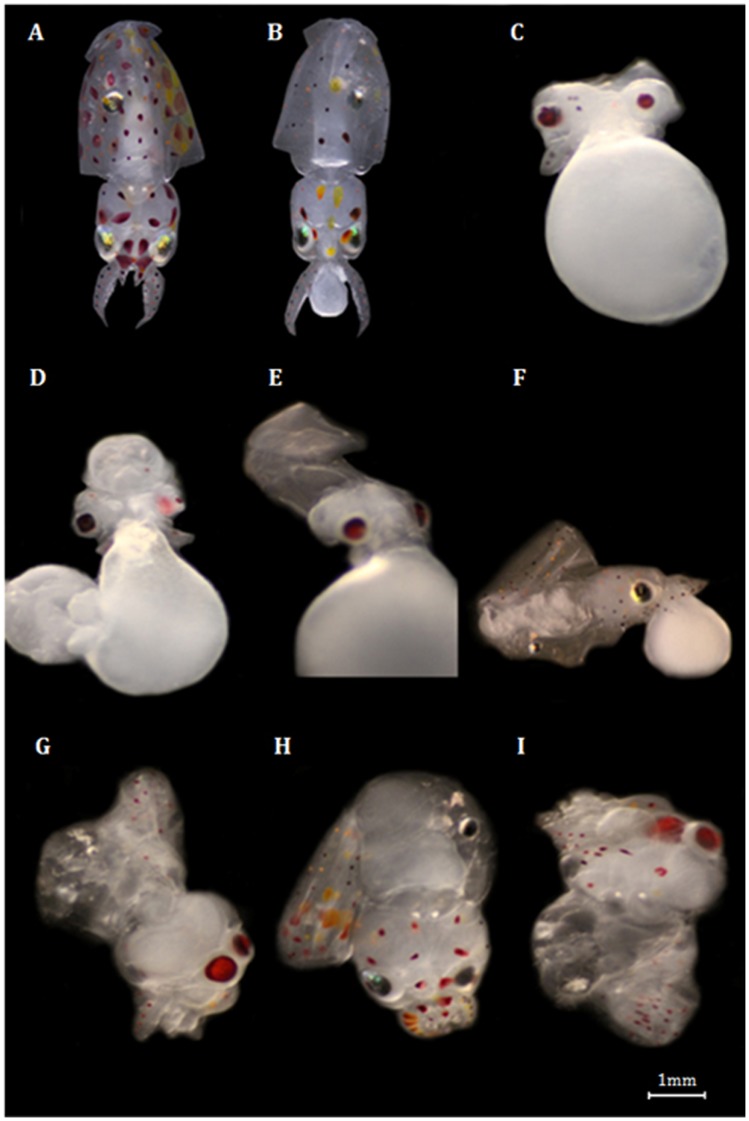
Abnormalities found during the early development squid, *Loligo vulgaris*. Panel A shows a “normal” hatchling and panel B a typical premature-hatchling. The most common abnormality types found in embryos: C) Eye dimorphism; D) Complete body deformity, E) Elongated body, F) Mantle deformity; and in hatchlings: G) Mantle detached; H) Mantle deformity; I) Complete body deformity.

**Figure 3:**
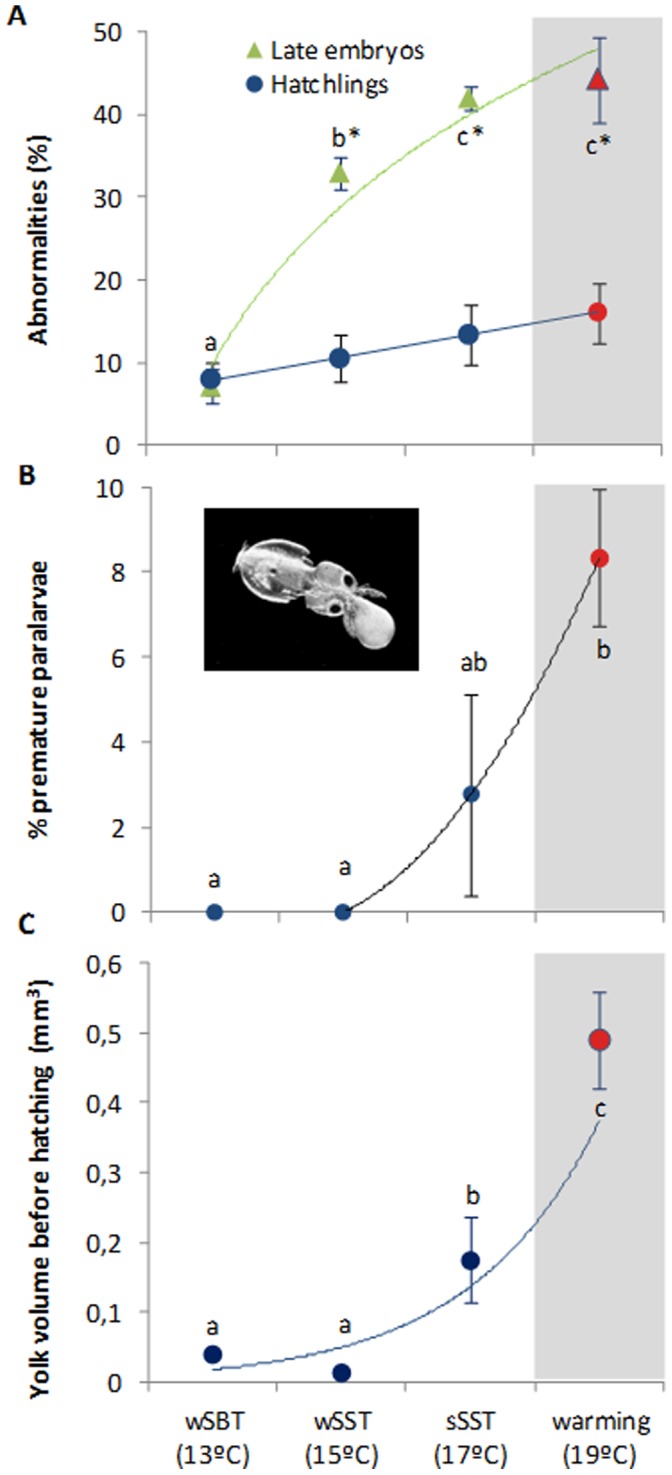
Abnormalities and premature hatching in the squid, *Loligo vulgaris*. Percent of total abnormalities (%) in late embryos and hatchlings (A), percentage of premature paralarvae (B), and yolk volume in late embryos (mm^3^)(C) at the different temperature scenarios (red symbols highlight the future summer scenario). Values are mean ± SD. Colored lines represent trendlines. In the upper panel, different letters and asterisks represent significant differences between temperatures and developmental stages, respectively (two way-ANOVA, more statistical details in Supporting Tables). In the middle and lower panels different letters represent significant differences (one-way ANOVA; more statistical details in Supporting Tables).

Future warming (19°C) significantly affected the early ontogenetic growth (Fig. 1C,D, Table S1). Embryo growth rates (expressed as % increase from beginning to the end of embryogenesis, and mm day^−1^) increased significantly under the present-day scenarios, but there was a significant trend reversal at future summer conditions (red symbols, 19°C, Fig. 1C,D, F = 92.3, p<0.001). Figure 2 shows the most common types of abnormalities observed. Early embryos showed mostly underdeveloped mantles, complete body deformities and eye dimorphism. Late embryos also showed elongated bodies and mantle deformities. Lastly, hatchlings revealed greater incidence of mantle detachment, mantle deformity and also complete body deformities. The incidence of abnormalities increased significantly with temperature and varied between stages; the higher percentage of abnormalities was found in late embryos exposed to the summer warming scenario (Fig. 3A, two-way ANOVA, p<0.001). Hatchlings presented the lower percentage of abnormalities in all thermal scenarios.

One of the most striking impacts of the future warming on squid early ontogeny was the enhancement of premature hatching (Fig. 3B,C). The percentage of paralarvae that hatched with the yolk-sac still attached was significantly greater at 19°C (red symbol, Fig. 3B, F = 7.2, p = 0.011), which was related to the fact that late embryos (pre-hatchlings) still had significant amount of yolk before hatching (Fig. 3C, F = 26.6, p<0.001).

### Metabolic Rates, Thermal Sensitivity and Anaerobic End-products

**Figure 4:**
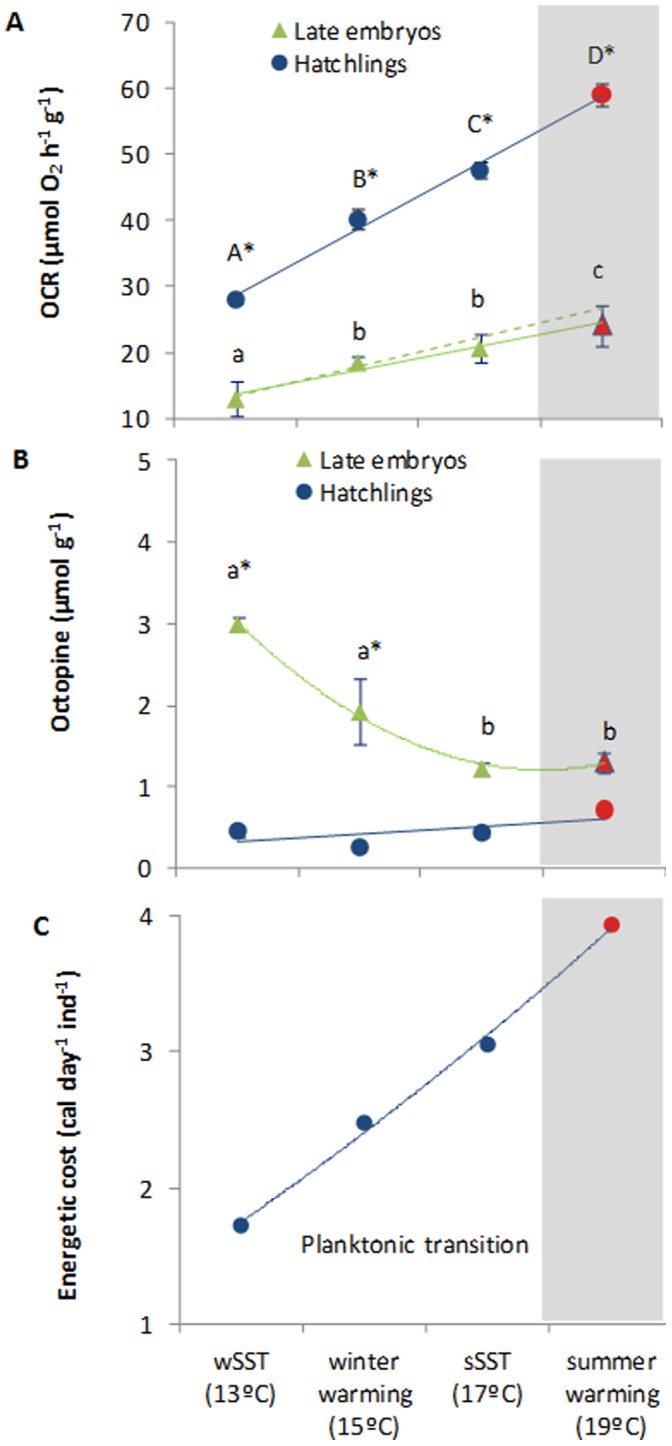
Metabolic physiology of squid (*Loligo vulgaris*) late embryos and hatchlings: A) oxygen consumption rates (µmolO_2_h^−1^g^−1^), B) octopine concentration (µmol g^−1^), and C) individual energetic cost of the planktonic transition (cal day^−1^ ind^−1^), at the different temperature scenarios (red symbols highlight the future summer scenario). Values are mean ± SD. Colored lines represent trendlines and different letters (capital letters for hatchlings; small letters for embryos) and asterisks represent significant differences between temperatures and developmental stages, respectively (more statistical details in Supporting Tables). In panel A, the “expected” trend is represented by the dash line assuming a Q_10_ of 2.5. In panel C, the energy-related values do not have any associated variance because they represent the metabolic augment (in calories, based on 4.7 kcal L^−1^ O2) from late embryos to planktonic paralarvae (i.e. using mean values from panel A).

**Figure 5:**
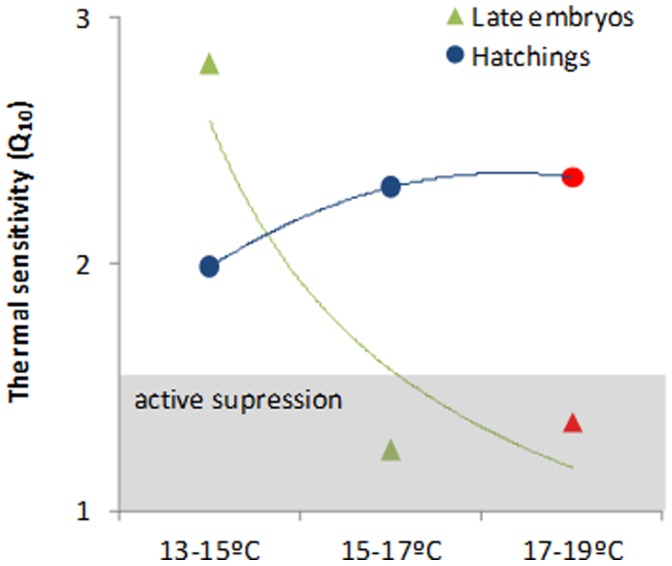
Thermal sensitivity (Q_10_) of late embryonic stages and hatchlings of *Loligo vulgaris* at the different temperature scenarios (red symbols highlight the future summer scenario). Coloured lines represent trendlines. Q_10_ values between 2 and 3 indicate active metabolic regulation; Q_10_ values inferior to 1.5 suggest active metabolic suppression.

Oxygen consumption rates (OCR) were significantly affected by temperature and developmental stage (Fig. 4A, two-way ANOVA, p<0.001). Late embryos displayed OCR ranging from 13.0 µmol O_2_ h^−1^g^−1^ at winter temperature (13°C) and 24.1 µmol O_2_ h^−1^g^−1^ at the summer warming condition (red symbol). Embryo’s Q_10_ values ranged around 1.5 (indicative of active metabolic suppression) above 15°C (Fig. 5). At normal operating temperatures, metabolic demand for oxygen increases with temperature with Q_10_ around 2–3 (this “expected” trend is represented by the dash line in Figure 4A).

Squid paralarvae displayed significantly higher OCR values than those observed for embryos, ranging from 28.1 µmol O_2_ h^−1^g^−1^ at winter temperature (13°C) and 59.1 µmol O_2_ h^−1^g^−1^ at the warming scenario (red symbol; Fig. 4A; Table S2). Contrary to late embryos, the Q_10_ values ranged always above 2 (Fig. 5). The energetic increment associated with the transition from encapsulated embryo to the planktonic life stage increased linearly with temperature (Fig. 4C).

Regarding the fermentative pathways, octopine levels were always higher in late embryos than in hatchlings. Moreover, the levels of this anaerobic end-product decreased significantly with increasing temperature in the former stages (Fig. 4B, two-way ANOVA, p<0.001). Such temperature effect was not observed in the latter stages (p>0.05).

### Thermal Tolerance Limits and Heat Shock Response

**Figure 6:**
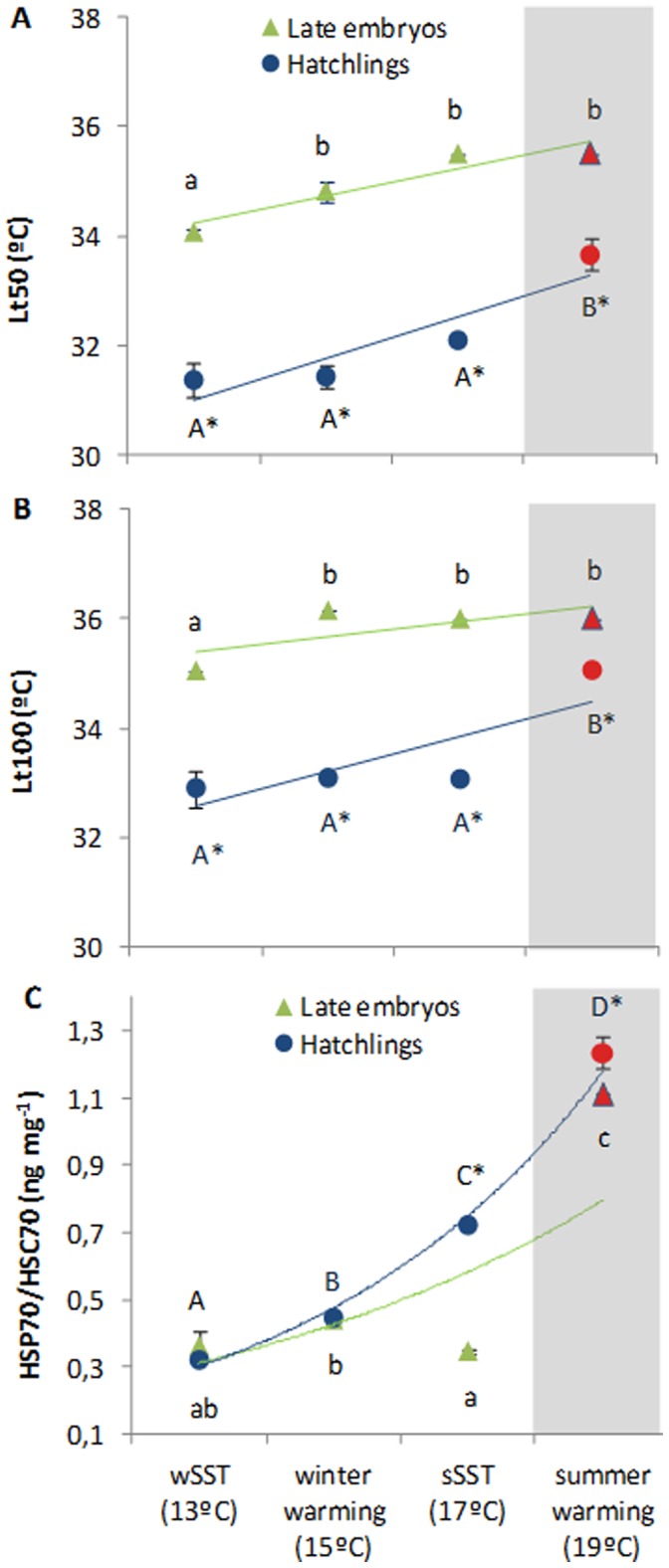
Thermal tolerance limits (A - Lt50, °C; B - Lt100, °C) and heat shock response (HSP70/HSC70, ng mg^−1^) (C) of late embryonic stages and hatchlings of the squid *Loligo vulgaris* at the different temperature scenarios (red symbols highlight the future summer scenario). Values are mean ± SD. Colored lines represent trendlines and different letters (capital letters for hatchlings; small letters for embryos) and asterisk represent significant differences between temperatures and developmental stages, respectively (more statistical details in Supporting Tables).

The thermal tolerance experiments revealed that the upper thermal tolerance limits were significantly affected by temperature and developmental stage (Fig. 6A and B, two-way ANOVA, p<0.001). Both LT_50_’s and LT_100_’s were positively influenced by the acclimation temperature, and such thresholds were significantly higher in late embryos than those observed for hatchlings (Fig. 6A and B; see linear-regression analyses Table S3). Concomitantly, there was a significant enhancement of the heat shock response (HSP70/HSC70) in both stages (Fig. 6C, two-way ANOVA, p<0.001), although stronger in the squid hatchlings (reaching 1.2 ng mg^−1^; red symbol). Although there was a significant temperature-stage interaction in heat shock response (F = 59.3, p<0.001; Table S2), there was no significant effect of acclimation in winter embryos (p<0.05).

### Oxidative Stress Tolerance

**Figure 7:**
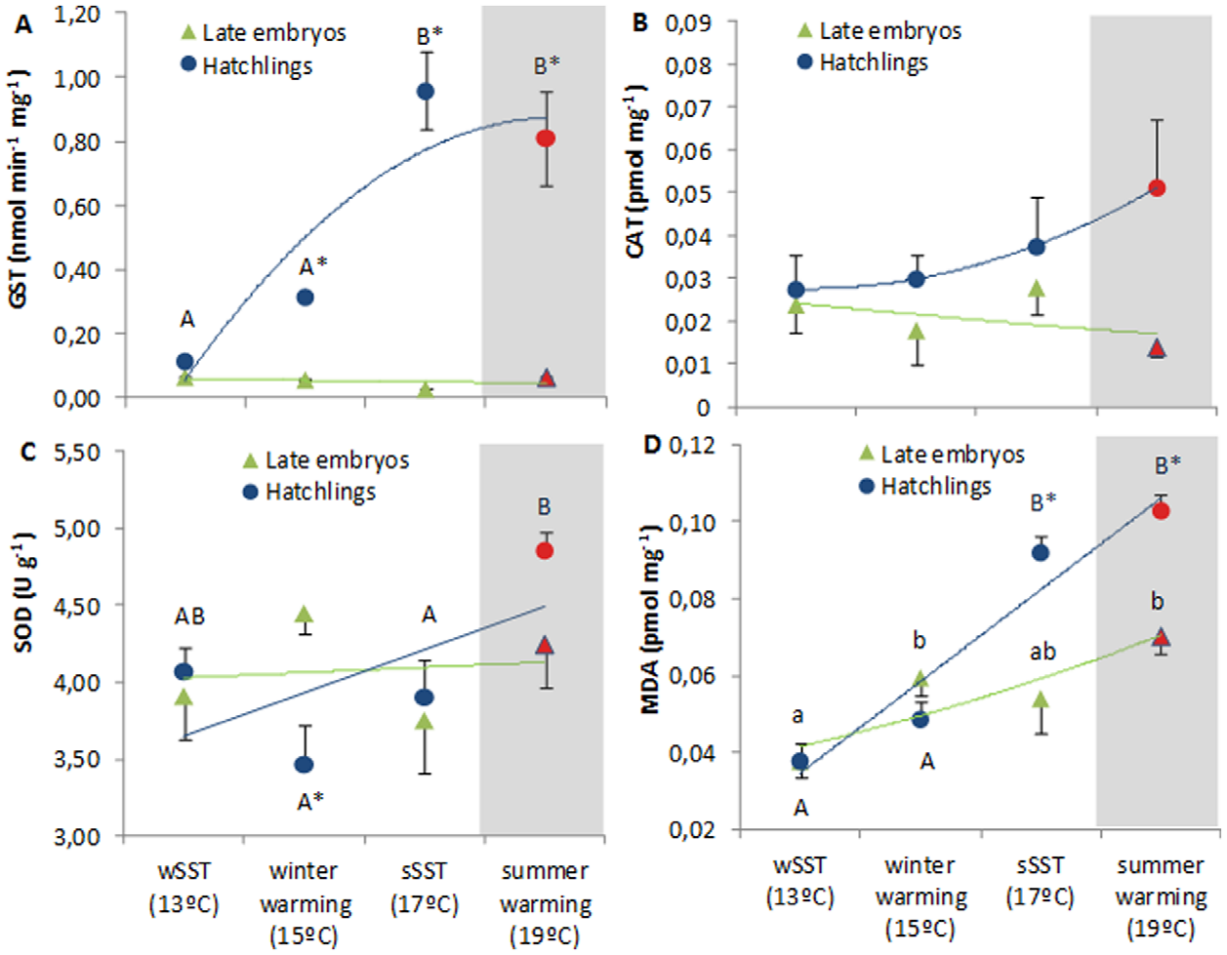
Antioxidant response of *Loligo vulgaris*’ late embryos and hatchlings at the different temperature scenarios: A) GST (nmol min^−1^mg^−1^), B) CAT (pmol mg^−1^), C) SOD (U g^−1^) and d) malondialdehyde (MDA; pmol mg^−1^). Red symbols highlight the future summer scenario. Values are mean ± SD. Colored lines represent trendlines and different letters (capital letters for hatchlings; small letters for embryos) and asterisk represent significant differences between temperatures and developmental stages, respectively (more statistical details in Supporting Tables).

Glutathione S-Transferase GST activity varied significantly between developmental stages, being significantly lower in the embryos (Fig. 7A, two-way ANOVA, p<0.001). Only the hatchlings revealed a significant positive temperature effect (p<0.05), but with a reversal pattern at 19°C (red symbol, Fig. 7A). Regarding catalase (CAT) activity, an opposite temperature effect between stages was observed (Fig. 7B, two-way ANOVA, p>0.05), with the hatchlings showing a non-significant positive trend. It is worth noting that CAT was the only variable analyzed that did not show a significant interaction between temperature and developmental stage (see also Table S2).

Superoxide dismutase (SOD) activity did not show any temperature-induced variation in late embryos. Yet, a significant increasing trend was observed in hatchlings (Fig. 7C, two-way ANOVA, p = 0.003), reaching 4.85 U g**^−^** ^1^ at the summer warming scenario (red symbol, Fig. 7C). Peroxidase activity (determined by the quantification of a specific end-product of the oxidative degradation process of lipids, the malondialdehyde - MDA) increased significantly with temperature in both developmental stages (Fig. 7D, two-way ANOVA, p<0.001). Such activity was more pronounced in hatchlings, especially in the summer warming scenario (0.1 pmol mg**^−^** ^1^; red symbol).

## Discussion

### Early Development under the Projected Near-future Ocean Warming

The embryonic phase in coastal (loliginid) squids represents a significant portion of the entire life-span and, compared to adults, developing embryos and hatchlings have quite limited behavioral plasticity and are passive recipients of their thermal environment [25], [26]. Here, we showed that the projected near-future ocean warming (only +2°C) is already above *L. vulgaris* optimal thermal tolerance boundaries, as embryonic development was dramatically shortened with a concomitant negative effect on growth and survival success (Fig. 1). Even though the underlying causes of embryonic death outside the range of normal development are not known, decreased membrane permeability, disequilibria of coupled enzyme reactions, and limits imposed by kinetics and inactivation of enzyme proteins could be some of the responsible mechanisms likely play a role [32].

The increased metabolic load incurred by squid embryos (Fig. 4A) resulted in lost growth potential (Fig. 1C,D), and after hatching, paralarvae were less developed (premature) and showed greater incidence of abnormalities (Fig. 3). Because energy absorbed from yolk is partitioned mainly between energy invested in newly formed tissue and energy expended in respiration [33], one can argue that at higher temperatures yolk-stores would be depleted more quickly and earlier in development. However, we showed that the percentage of paralarvae that hatched with the yolk-sac still attached was significantly higher and that the pre-hatchlings still had significant amount of yolk before hatching (Fig. 3 B,C). We suggest that the acceleration of the hatching process was mainly promoted by the higher oxygen demands coupled with hypoxic stress (see below). In fact, premature hatching is a common response to hypoxia in fish [34].

### Hypoxia and Metabolic Suppression in Embryos

As expected, increased temperatures led to higher embryonic metabolic rates (Fig. 4A). To increase the flow of oxygen by means of diffusion, invertebrate eggs swell, leading to greater surface areas and reduced egg wall thicknesses [35]. Yet, such swelling does not prevent *p*O_2_ from consistently falling (to critical levels) and *p*CO_2_ from rising within cephalopod eggs [36], [37]. Here, the elevated catabolic activity, and consequently OCRs, seemed to have accelerated hypoxia within egg capsules, and caused metabolic suppression. This finding was corroborated with thermal sensitivity data, which revealed that late embryos displayed Q_10_ values below 1.5, indicative of active metabolic suppression [38]. Concomitantly, and regardless of temperature, the fermentative glucose-opine pathway (end-product: octopine) was more used by embryos (Fig. 4B). This may be linked to the reduced capacity to extract oxygen at hypoxic and hypercapnic conditions within eggs [39]. These stressful abiotic conditions inside eggs are expected to be aggravated under the projected near-future ocean warming, with deleterious effects on embryo’s survival and growth.

### Feeding Challenges Faced by Jet-propelled Hatchings

Most marine invertebrates have a distinct larval phase in their early life histories and can be divided into species whose larvae feed in the plankton (planktotrophic; e.g. squids) and species whose larvae can develop and metamorphose without feeding (lecithotrophic: e.g. abalones; echinoderms) [19], [40]. Thus, the impact of warming in the early ontogeny will also greatly depend on the type of developmental modes within and among taxa. After hatching, the planktonic squid paralarvae rely predominantly on a pulsed jet for locomotion [41], which distinguishes them from the majority of aquatic locomotors that employ mostly oscillatory/undulatory movements [6], [42]. So, the energy losses due to swimming activity, which account for a large proportion of the total squid energy budget, are expected to increase with warming. Here, we showed that the “metabolic burst” associated with the transition from an encapsulated embryo to a jet propelled planktonic stage increased linearly with temperature (Fig. 4C). Therefore, the jet-propelled squid hatchlings will require more food per unit body size at higher temperatures and feeding failures will be critical since they show high metabolic rates and low levels of metabolic reserves [25]. The time period that new hatchlings can survive without food is known to be very limited at higher temperatures [25] and so, they will need more food but also have less time to find it before facing mortality.

### Thermal Tolerance Limits

Available knowledge of mechanistic principles shows that thermal windows are narrow in all early life stages, due to developmental constraints and insufficient capacity of central organs [5], [13]. In the present study, we found significant differences in the thermal tolerance (LT50 and LT100) between winter and summer embryos, with the latter revealing to be better adapted to warming than the former. This was supported by the greater heat shock response in the summer embryos (Fig. 6C). Parental effects may have played a critical role on such trends since winter spawners tend to show higher fecundity and smaller oocytes. This strategy favors higher number of offspring surviving natural mortality, rather than higher individual fitness in the colder periods [24]. Thus, the extent to which these distinct spawning cohorts are adapted to the prevailing temperatures may have implications for the population survival and genetic composition under this rapidly changing thermal regime.

The few thermal-related LT50 studies on early stages suggest that thermal tolerance range of embryos is lower than those found in larvae, because once larvae hatch, the temperature range over which they can persist increases significantly [43]. Here, independent of the thermal environment, the embryo was more heat-tolerant (Fig. 6A,B). The lack of physical protection provided by the egg masses after hatching may contribute to the lower thermal tolerance of the hatchlings. Also, their lower thermal tolerance limits may be related to the high oxygen demands associated with the planktonic life strategy, which is coupled with an inefficient mode of locomotion (see previous section). This statement is supported by the concept that links thermal tolerance windows directly to oxygen supply and energy demand, i.e., “oxygen-limited thermal tolerance” (OLTT) hypothesis [5], [13]. This concept implies that oxygen supply to tissues is optimal between limits called lower and upper pejus temperatures. Thus, it is expected that one of the major limitations that the squid hatchlings will face in the future warming scenario (19°C) is the ability to extract enough oxygen from the water to match the demand dictated by biochemical processes. Yet, the thermal tolerance limits of the paralarvae increased with temperature (Fig. 6A,B). This does not support the OLTT hypothesis that argues that organisms facing higher oxygen demand should exhibit decreased thermal tolerance (see the positive correlations in Supporting Information). Besides the possible influence of parental effects [33], the squid early stages could have supported higher metabolic rates by adjusting mitochondrial densities and functional properties in order to increase their thermal tolerance windows [44]. By altering the lipid bilayer composition of the mitochondria, the types of enzymes/isoforms present, and membrane protein composition, squids may enhance the efficiency of respiration in the mitochondria and therefore change their metabolic demand for oxygen in order to match the supply by the environment.

### Heat Shock Response, Cell Damage and Oxidative Stress Tolerance Strategies

The greater exposure to environmental stress by the hatchlings seems to be compensated by physiological mechanisms that reduce stress negative effects on fitness. The HSC70/HSP70 concentrations were greatest in the near-future warming scenario for both developmental stages, with hatchlings showing the largest increase (Fig. 6C). The increased metabolic demands faced by the hatchlings must lead to elevated ROS formation, and HSPs are among the molecules that can eliminate/change the molecular configuration of ROS [45]. Concomitantly, warming also led to an augment of MDA concentrations (see correlative values in Table S4), indicative of the enhancement of ROS action in organism’s lipids (“peroxidation”), a process considered to be one of the most frequent cellular injury mechanisms [30].

The antioxidative enzymes are known to be intrinsically linked and dependent upon the activity of one another, and therefore, one would expect to see correlative changes in their activity [46]. Here, this combined effect was evident in the hatchlings but absent in the embryos (Fig. 7A,B,C; see correlations in Table S5). In fact, in the projected summer warming scenario, the increased oxygen requirements of the hatchlings led to higher SOD activity. This indicates that there was an increase of superoxide production. Concomitantly, CAT activity also was enhanced possibly to increment the capability to catabolize peroxide resulting from SOD action. Our results suggest that heat shock proteins, SOD and CAT production constituted an integrated stress response in the squid paralarvae, but not in embryos, to ocean warming.

In conclusion, ocean warming is expected to drive profound biological impacts on marine biota, yet most research has been conducted on adult stages, although early stages are assumed to be the most vulnerable. We argue that the stressful abiotic conditions inside squid eggs will be aggravated under the projected near-future ocean warming, with deleterious effects on embryo’s survival and growth. The greater feeding challenges and the lower thermal tolerance limits of the hatchlings will be connected to the high metabolic demands associated with the planktonic life strategy. In the future, the early stages might support higher energy demands by enhancing physiological mechanisms that reduce negative effects on fitness and adjusting some functional properties to increase their thermal tolerance windows.

## Materials and Methods

### Egg Collection and Incubation

Recently-spawned egg masses (eggs with cleavage and formation of germinal layers; stage I-V [47]) were collected in Figueira da Foz and Cascais (Western coast of Portugal) by commercial vessels (between 36–54 m depth) in the winter and summer periods of 2011, respectively. After collection, eggs were immediately transferred to the aquaculture facilities in Laboratório Marítimo da Guia, Cascais. Egg strands were placed in four recirculating systems, each containing 25 separate glass aquariums (volume of 54 L) and a collective sump of 270 L. The closed systems were filled with UV-sterilized and filtered (series 20, 10, 5 and 1 µm) seawater and tanks were placed on a with a photoperiod of 14-h light:10-h dark cycle. Water quality was mantained using wet-dry filters, protein skimmers (Schuran, Jülich, Germany) and 30W UV-sterilizers (TMC, Chorleywood, UK). Ammonia and nitrite were monitored regularly and kept below detectable levels. Salinity was kept at 34.0±1.0 and pH was maintained at 8.1±0.1. Temperature was regulated via Heilea chillers (Guangdong, China). Egg strands were suspended 5–15 cm below the water surface, to ensure good aeration. To take into account the influence early life history via the thermal regimes experience by the progeny, eggs were collected in cooler (winter) and warmer (summer) periods. It is worth noting that one important feature in squid spawning is that each mass has eggs from multiple mothers. Winter egg masses were reared at: i) 13°C - the mean sea surface temperature in winter (wSST) in western coast of Portugal [23], and ii) 15°C – the expected winter warming scenario in 2100 (+2°C; [31]). The summer egg masses were reared at: i) 17°C – the mean sea surface summer temperature (sSST), and ii) 19°C – the future sSST warming scenario for the western coast of Portugal in 2100 (+2°C; [31]). Ten egg masses were incubated per each thermal treatment.

### Survival, Growth and Abnormalities

In the beginning of the experiment, 45 embryos were randomly individualized from the 10 egg masses in three independent boxes (with 15 slots each) for each temperature, and were followed until hatchlings (paralarval stage). The initial size varied around 1.72 mm±0.17 for the recently spawned winter embryos and around 2.07±0.19 for the summer embryos. Each embryo was observed at 48-hr intervals using a dissecting microscope to follow growth and survival rates. No differences were expected due to the handling [48]. In each observation, developmental stage was identified and the following measurements were made, namely: egg length and width, yolk-sac length and width, and embryo length (from the moment that yolk-sac and embryo were well differentiated). Yolk-sac measurements were used to determine yolk volume, using the following formulas: i) for oblate(spheroid)-shaped yolk-sacs - V = 1/6(W^2^L); ii) for spheroid-shaped ones - V = 4/3(R^3^) [(V = volume, W = width, L = length, R = radius)].

From the egg masses incubated at the each thermal scenario, one hundred late embryos and two hundred hatchlings were screened for abnormalities. Abnormalities were defined as anomalous growth in the body shape, such as underdeveloped mantle, mantle detached, eye dimorphism, elongated body, complete body deformity, among others (see [48]). For each temperature, the percentage of individuals with abnormalities was determined. Premature larvae were also taken into consideration, being defined as the paralarvae that hatched with the yolk-sac still attached.

### Oxygen Consumption Rates and Thermal Sensitivity

Oxygen consumption measurements were determined according to previously established methods [49]. Late eggs (pre-hatching) and paralarvae (hatchlings) were incubated in sealed water-jacketed respirometry chambers (RC300 Respiration cell, Strathkelvin, North Lanarkshire, Scotland) containing filtered seawater mixed with antibiotics (50 mg L**^−^** ^1^ streptomycin) to avoid bacterial respiration. Water volumes were adjusted in relation to animal mass (up to 4 mL) in order to minimize locomotion and stress but still allow spontaneous and routine activity rates of the hatchlings. Bacterial controls (blanks) were conducted to correct for possible bacterial respiratory activity. Respiration chambers were placed in water baths (Lauda, Lauda-Königshofen, Germany) to control temperature. Oxygen concentrations were recorded with Clarke-type O_2_ electrodes connected to a multi-channel oxygen interface (Strathkelvin, North Lanarkshire, Scotland). The duration of respiratory runs varied from 12 to 24 h. Thermal sensitivity (Q_10_) was determined using the standard equation:
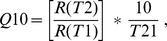
where *R*(*T*_2_) and *R*(*T*_1_) represent the oxygen consumption rates at temperatures *T*_2_ and *T*_1_, respectively.

### Thermal Tolerance Limits

The upper thermal tolerance limits were determined based on Stillman and Somero [11]. Sixty late embryos and sixty squid paralarvae were incubated in small containers with approximately 100 mL of seawater taken from the rearing aquarium. Each container had 20 specimens (late embryos or paralarvae) comprising 3 replicates (total n = 60). These glass containers were suspended in a temperature regulated water bath that was controlled to the nearest 0.1°C. The temperature of the water bath was set to the acclimation temperature and maintained for 30 min. Thereafter, the temperature was increased at a rate of 1°C/30 min. Every 30 min, the water was aerated with an air stone and the temperature in each container was checked (with thermocouple probes), and the swimming activity (i.e. jet propulsion) and mantle contractions (when at the bottom) of each paralarvae was visually monitored. If no responsiveness was noticed, the specimen was considered to be dead. The percentage of living individuals at each temperature was calculated and then transformed by the arcsine square root function and expressed in radians. Linear-regression analysis was then used to find the slope of the line, from which the temperature at which 50% of the organisms had died (0.785 radians) was calculated. This was used as the measure for upper thermal tolerance limits and referred to as the LT50. The LT100 (temperature at which all specimens were dead) was also recorded in the end.

### Biochemical Analyses

#### Octopine determination

Octopine (anaerobic metabolite) is oxidized to pyruvate and arginine by octopine dehydrogenase in the presence of NAD+. The increase in NADH concentration, measured by the change in the absorbance at 339 nm (Shimadzu, UV-1800), is proportional to the amount of octopine [44]. The method entailed the preparation of perchloric acid (3 M) extracts from late embryos and paralarvae and determinations were made immediately after neutralization with KHCO_3_.

#### Preparation of tissue extracts for heat shock proteins and antioxidative enzymes

Homogenates were prepared, in triplicate, by using 150 mg of frozen tissue of both stages. To obtain enough tissue, 50 specimens (late embryos and hatchlings) were homogenized for each replicate sample. The pooled tissues were homogenized in 500 µL of the homogenization buffer, Phosphate Buffer Saline buffer solution (PBS, pH 7.3*)*: 0.14 M NaCl, 2.7 mM KCl, 8.1 mM Na_2_HP0_4_, 1.47 mM KH_2_P0_4_) by using a glass hand held homogenizer. All homogenates were then centrifuged (20 min at 14 000×g at 4°C) and enzyme activities and heat shock proteins were measured in the supernatant fraction. HSR was assessed from HSP70/HSC70 expression (see below). All the samples were analyzed for Glutathione S-Transferase, catalase, superoxide dismutase, lipid peroxidase activity and Hsp70 levels. All enzyme assays were tested with commercial enzymes obtained from Sigma (St. Louis, USA).

#### Heat shock response (HSP70/HSC70)

HSP70/HSC70 content was assessed by ELISA (Enzyme-Linked Immunoabsorbent Assay) adapting a protocol from Njemini et al. [50]. Briefly, 10 µL of the homogenate’s supernatant was diluted in 250 µL of PBS (1x), and 50 µL of the diluted sample was added to a 96 well microplates (Nunc-Roskilde, Denmark) and allowed to incubate overnight at 4°C. In the next day the microplates were washed (3X) in 0.05% PBS-Tween-20. One hundred µL of blocking solution (1% BSA, Bovine Serum Albumin, Sigma-Aldrich, USA) was added to each well and left to incubate at room temperature for 2 hours. After microplates wash, 50 µL of 5 µg mL**^−^** ^1^ primary antibody (anti-HSP70/HSC70, Acris USA), detecting 72 and 73 kDa proteins corresponding to the molecular mass of inducible hsp and hsc70, was added to each well and then incubated at 37°C for 90 min. The non-linked antibody was removed by washing the microplates again, which were then incubated for 90 min at 37°C with 50 µL of 1 µg mL**^−^** ^1^ of the secondary antibody, anti-mouse IgC, Fab specific, alkaline phosphatase conjugate, Sigma-Aldrich, USA. After another wash, 100 µL of substrate (SIGMA *FAST*™ p-Nitrophenyl Phosphate Tablets, Sigma-Aldrich, USA) was added to each well and incubated 10–30 min at room temperature. Fifty µL of stop solution (3N NaOH) was added to each well and then the absorbance read at 405 nm in a 96 well microplate reader (BIO-RAD, Benchmark, USA). The amount of Hsp70/Hsc70 in samples was calculated from a curve of absorbance based on serial dilutions of purified HSP70 active protein (Acris, USA) to give a range from 0 to 2000 ng/mL. The results were expressed in relation to wet weight of the sample (ng hsp70/hsc70 mg^−1^ ww).

#### Glutathione S-Transferase

The enzyme activity was determined spectrophotometrically in the supernatant at 340 nm, every minute for 6 minutes using a microplate reader (BIO-RAD, Benchmark, USA). The assay contained 200 mM L-glutathione reduced, Dulbecco’s Phosphate Buffered Saline and 100 mM CDNB (1-Chloro-2,4-Dinitrobenzene Solution). Equine liver GST (Sigma-Aldrich, Germany) was used as standard and positive control. The increase in absorbance is directly proportional to the GST activity. The results are expressed in relation to wet weight of the sample (nmol min^−1^mg^−1^ ww).

#### Catalase

The assay contained a total reaction volume of 3 mL composed by 50 mmol l^−1^ potassium phosphate buffer (pH 7.0), 12.1 mmol l^−1^ H_2_O_2_ as a substrate and the reaction was started by the addition of the sample. The consumption of peroxide (extinction coeff. 0.04 mmol^−1^cm^−1^) was monitored using a spectrophotometer Helios (Unicam, UK) at 240 nm and 25°C each 15 seconds during 180 seconds. Standard catalase activity was measured using a bovine catalase solution (Sigma-Aldrich, Germany) of 1523.6 U/mL. The results are expressed in relation to the wet weight of the sample (pmol min^−1^ mg^−1^ ww).

#### Superoxide dismutase (SOD)

SOD activity was determined spectrophotometrically in the supernatant at 25°C (BIO-RAD, Benchmark, USA) at 550 nm. The adapted assay contained, at 25°C, 50 mM Potassium Phosphate Buffer (pH 7.8), 3 mM EDTA, 3 mM Xantine solution, 0.75 mM NBT (nitroblue tetrazolium), 100 mU XOD (Xanthine Oxidase Solution) and 1 U/µL SOD Enzyme solution. SOD from bovine erythrocytes (Sigma-Aldrich, Germany) was used as standard and positive control. The results of this enzymatic assay are given in units of SOD activity per milligram of wet weight of the sample (U mg**^−^** ^1^ ww), where one unit of SOD is defined as the amount of sample causing 50% inhibition of NBT reduction.

#### Lipid peroxidase (determination of malondialdehyde, MDA)

Lipid peroxidase activity was determined by the quantification of a specific end-product of the oxidative degradation process of lipids, the malondialdehyde (MDA). TBARS Assay (thiobarbituric acid reactive substances assay) was used, in which thiobarbituric acid reacts with MDA to yield a fluorescent product that was detected spectrophotometrically at 532 nm. Homogenates were treated with 8.1% dodecyl sulfate sodium, 20% trichloroacetic acid (pH 3.5), thiobarbituric acid, mixture of n-butanol and pyridine (15∶1, v/v) (Sigma-Aldrich, Germany). To quantify the lipid peroxides, MDA concentrations were calculated with the computer program Microplate Manager 4.0 (BIO-RAD, USA) based on an eight-point calibration curve (0–0.3 µM TBARS) using MDA bis (dimethyl acetal) (from Merck). The results were expressed in relation to wet weight of the sample (pmol mg^−1^ ww).

### Statistical Analyses

Pearson’s correlation coefficients were used to get an indication of the relationships among the following variables: oxygen consumption rates, thermal tolerance limits, heat shock protein response and antioxidative enzyme activities. Two-way ANOVAs were conducted to detect significant differences in abnormalities, oxygen consumption rates, thermal tolerance limits (LT50 and Lt100), HSP70/HSC70, GST, CAT, SOD and MDA contents between temperature and developmental stage (late embryos and hatchlings). Additionally, one-way ANOVA was used to evaluate the effect of temperature on development time, survival rates, paralarvae length, embryo growth increment, embryo growth, premature paralarvae, yolk volume before hatchling. Previously, normality and homogeneity of variances were verified by Kolmogorov– Smirnov and Bartlett tests, respectively. Moreover, percentage data (% survival, % abnormalities and % premature paralarvae) was previously transformed by arc sine square root function. Subsequently, post-hoc tests (Tukey HSD and unequal N HSD) were performed. All statistical analyses were performed for a significance level of 0.05, using Statistica 10.0 software (StatSoft Inc., Tulsa, USA).

## Supporting Information

Table S1 **Results of one-way ANOVA evaluating the effect of temperature on development time, survival rates, embryo growth increment, embryo growth, premature paralarvae, yolk volume before hatchling in squid (** ***Loligo vulgaris*** **) embryos and hatchlings.**
(DOCX)

Table S2 **Results of two-way ANOVA evaluating the effects of temperature and** ***Loligo vulgaris*** **developmental stage (late embryos and hatchlings) on abnormalities, oxygen consumption rates, octopine concentration, thermal tolerance limits (LT50 and Lt100), and HSP70/HSC70, GST, CAT, SOD and MDA contents.**
(DOCX)

Table S3 **Results from linear-regression analysis to measure the upper thermal tolerance limits (LT 50 and LT 100) in the late embryos and paralarvae of** ***Loligo vulgaris.*** Note: the percentage of alive at each temperature was calculated and then transformed by the arcsine square root function and expressed in radians.
(DOCX)

Table S4 **Pearson correlation coefficients between oxygen consumption rates (RMR) and thermal tolerance limits (LT50 and LT100) in the late embryos and paralarvae of** ***Loligo vulgaris.***
(DOCX)

Table S5 **Pearson correlation coefficients between oxygen consumption rates (OCR), heat shock protein 70 (HSP70/HSC70), catalase (CAT), superoxide-dismutase (SOD), glutathione S-transferase GST and malondialdehyde (MDA) concentrations in the paralarvae of** ***Loligo vulgaris.***
(DOCX)
