# Supplementary material for: Ocean Warming Enhances Malformations, Premature Hatching, Metabolic Suppression and Oxidative Stress in the Early Life Stages of a Keystone Squid
Source: PLoS One. 2012 Jun 6;7(6):e38282. doi: 10.1371/journal.pone.0038282 (PMC3368925; doi:10.1371/journal.pone.0038282)
Supplement: Table S1 — Results of one-way ANOVA evaluating the effect of temperature on development time, survival rates, embryo growth increment, embryo growth, premature paralarvae, yolk volume before hatchling in squid ( Loligo vulgaris ) embryos and hatchlings. (DOCX) [file pone.0038282.s001.docx]

**Supporting Information**

Table S1 - Results of one-way ANOVA evaluating the effect of temperature on development time, survival rates, embryo growth increment, embryo growth, premature paralarvae, yolk volume before hatchling in squid (*Loligo vulgaris*) embryos and hatchlings.

|  |  |  |  |  |  |
| --- | --- | --- | --- | --- | --- |
|  | | **df** | **MS** | **F** | **p** |
| **Development time** | |  |  |  |  |
| *Temperature (T)* | | 3 | 817.4 | 459.0 | 0.000 |
| *Error* | | 133 | 1.8 |  |  |
|  | |  |  |  |  |
| **Survival** | |  |  |  |  |
| *Temperature (T)* | | 3 | 0.229 | 4.229 | 0.046 |
| *Error* | | 8 | 0.054 |  |  |
|  | |  |  |  |  |
| **Embryo growth increment** | |  |  |  |  |
| *Temperature (T)* | | 3 | 224,540 | 129.8 | 0.000 |
| *Error* | | 133 | 1730 |  |  |
|  | |  |  |  |  |
| **Embryo growth** | |  |  |  |  |
| *Temperature (T)* | | 3 | 0.1 | 92.3 | 0.000 |
| *Error* | | 133 | 0.0 |  |  |
|  | |  |  |  |  |
| **Yolk volume before hatching** | |  |  |  |  |
| *Temperature (T)* | | 3 | 1.1 | 26.6 | 0.000 |
| *Error* | | 133 | 0.0 |  |  |
|  | |  |  |  |  |
| **Premature paralarvae** | |  |  |  |  |
| *Temperature (T)* |  | 3 | 0.056 | 7.2 | 0.011 |
| *Error* |  | 133 | 0.007 |  |  |
