# Supplementary material for: Ocean Warming Enhances Malformations, Premature Hatching, Metabolic Suppression and Oxidative Stress in the Early Life Stages of a Keystone Squid
Source: PLoS One. 2012 Jun 6;7(6):e38282. doi: 10.1371/journal.pone.0038282 (PMC3368925; doi:10.1371/journal.pone.0038282)
Supplement: Table S2 — Results of two-way ANOVA evaluating the effects of temperature and Loligo vulgaris developmental stage (late embryos and hatchlings) on abnormalities, oxygen consumption rates, octopine concentration, thermal tolerance limits (LT50 and Lt100), and HSP70/HSC70, GST, CAT, SOD and MDA contents. (DOCX) [file pone.0038282.s002.docx]

**Supporting Information**

Table S2 - Results of two-way ANOVA evaluating the effects of temperature and *Loligo vulgaris* developmental stage (late embryos and hatchlings) on abnormalities, oxygen consumption rates, octopine concentration, thermal tolerance limits (LT50 and Lt100), and HSP70/HSC70, GST, CAT, SOD and MDA contents.

|  | **df** | **MS** | **F** | **p** |
| --- | --- | --- | --- | --- |
| **Abnormalities** |  |  |  |  |
| *Temperature (T)* | 3 | 0.088 | 85.4 | 0.000 |
| *Stage (S)* | 1 | 0.335 | 32407 | 0.000 |
| *T x S* | 3 | 0.043 | 41032 | 0.000 |
| *Error* | 24 | 0.001 |  |  |
|  |  |  |  |  |
| **OCR** |  |  |  |  |
| *Temperature (T)* | 3 | 424.1 | 332.8 | 0.000 |
| *Stage (S)* | 1 | 3792.6 | 2976.1 | 0.000 |
| *T x S* | 6 | 88.6 | 69.5 | 0.000 |
| *Error* | 60 | 1.3 |  |  |
|  |  |  |  |  |
| **Octopine** |  |  |  |  |
| *Temperature (T)* | 3 | 0.9 | 12.3 | 0.000 |
| *Stage (S)* | 1 | 11.6 | 155.2 | 0.000 |
| *T x S* | 3 | 1.2 | 16.1 | 0.000 |
| *Error* | 16 | 0.1 |  |  |
|  |  |  |  |  |
| **Lt50** |  |  |  |  |
| *Temperature (T)* | 3 | 3.9 | 59.2 | 0.000 |
| *Stage (S)* | 1 | 40.5 | 715.1 | 0.000 |
| *T x S* | 3 | 0.9 | 12.3 | 0.000 |
| *Error* | 16 | 0.1 |  |  |
|  |  |  |  |  |
| **Lt100** |  |  |  |  |
| *Temperature (T)* | 3 | 2.5 | 39.0 | 0.000 |
| *Stage (S)* | 1 | 30.9 | 488.8 | 0.000 |
| *T x S* | 3 | 1.4 | 22.4 | 0.000 |
| *Error* | 16 | 0.0 |  |  |
|  |  |  |  |  |
| **GST** |  |  |  |  |
| *Temperature (T)* | 3 | 225,171 | 33.5 | 0.000 |
| *Stage (S)* | 1 | 1470,897 | 219.2 | 0.000 |
| *T x S* | 3 | 253,818 | 37.8 | 0.000 |
| *Error* | 16 | 6,712 |  |  |
|  |  |  |  |  |
| **SOD** |  |  |  |  |
| *Temperature (T)* | 3 | 0.62 | 7.2 | 0.003 |
| *Stage (S)* | 1 | 0.00 | 0.0 | 0.885 |
| *T x S* | 3 | 0.69 | 8.0 | 0.002 |
| *Error* | 16 | 0.09 |  |  |
|  |  |  |  |  |
| **MDA** |  |  |  |  |
| *Temperature (T)* | 3 | 0.003 | 67.9 | 0.000 |
| *Stage (S)* | 1 | 0.001 | 33.0 | 0.000 |
| *T x S* | 3 | 0.001 | 21.4 | 0.000 |
| *Error* | 16 | 0.000 |  |  |
|  |  |  |  |  |
| **HSP70/HSC70** |  |  |  |  |
| *Temperature (T)* | 3 | 0.84 | 959.6 | 0.000 |
| *Stage (S)* | 1 | 0.08 | 91.1 | 0.000 |
| *T x S* | 3 | 0.05 | 59.3 | 0.000 |
| *Error* | 16 | 0.00 |  |  |
|  |  |  |  |  |
| **CAT** |  |  |  |  |
| *Temperature (T)* | 3 | 0.000 | 0.431 | 0.732 |
| *Stage (S)* | 1 | 0.003 | 6.898 | 0.013 |
| *T x S* | 3 | 0.001 | 1.398 | 0.260 |
| *Error* | 34 | 0.000 |  |  |
