# Supplementary material for: Ocean Warming Enhances Malformations, Premature Hatching, Metabolic Suppression and Oxidative Stress in the Early Life Stages of a Keystone Squid
Source: PLoS One. 2012 Jun 6;7(6):e38282. doi: 10.1371/journal.pone.0038282 (PMC3368925; doi:10.1371/journal.pone.0038282)
Supplement: Table S3 — Results from linear-regression analysis to measure the upper thermal tolerance limits (LT 50 and LT 100) in the late embryos and paralarvae of Loligo vulgaris. Note: the percentage of alive at each temperature was calculated and then transformed by the arcsine square root function and expressed in radians. (DOCX) [file pone.0038282.s003.docx]

**Supporting Information**

Table S3. Results from linear-regression analysis to measure the upper thermal tolerance limits (LT 50 and LT 100) in the late embryos and paralarvae of *Loligo vulgaris.* Note: the percentage of alive at each temperature was calculated and then transformed by the arcsine square root function and expressed in radians.

| **Treatment** |  | **LT50** | **LT100** | **R^2^** | **F** | **p** | **Equation** |
| --- | --- | --- | --- | --- | --- | --- | --- |
|  |  |  |  |  |  |  |  |
| Late embryos |  |  |  |  |  |  |  |
| 13ºC | Replicate 1 | 34,0 | 35,0 | 0,9946 | 182,6 | 0,047 | y = -1.2663x + 35.037 |
|  | Replicate 2 | 34,1 | 35,1 | 0,9643 | 27,0 | 0,121 | y = -1.2278x + 35.071 |
|  | Replicate 3 | 34,1 | 35,1 | 0,9876 | 79,7 | 0,071 | y = -1.2575x + 35.051 |
|  |  |  |  |  |  |  |  |
| 15ºC | Replicate 1 | 34,8 | 36,1 | 0,9784 | 45,2 | 0,094 | y = -1.5666x + 36.062 |
|  | Replicate 2 | 35,1 | 36,1 | 0,9643 | 27,0 | 0,121 | y = -1.2278x + 36.071 |
|  | Replicate 3 | 34,5 | 36,3 | 0,8554 | 17,7 | 0,024 | y = -2.2793x + 36.324 |
|  |  |  |  |  |  |  |  |
| 17ºC | Replicate 1 | 35,5 | 36,0 | 0,9999 | 18503,0 | 0,005 | y = -0.6366x + 36.000 |
|  | Replicate 2 | 35,5 | 36,0 | 0,9999 | 18503,0 | 0,005 | y = -0.6366x + 36.000 |
|  | Replicate 3 | 35,5 | 36,0 | 0,9999 | 18503,0 | 0,005 | y = -0.6366x + 36.000 |
|  |  |  |  |  |  |  |  |
| 19ºC | Replicate 1 | 35,5 | 36,0 | 0,9999 | 18503,0 | 0,005 | y = -0.6366x + 36.000 |
|  | Replicate 2 | 35,5 | 36,0 | 0,9999 | 18503,0 | 0,005 | y = -0.6366x + 36.000 |
|  | Replicate 3 | 35,5 | 36,0 | 0,9999 | 18503,0 | 0,005 | y = -0.6366x + 36.000 |
|  |  |  |  |  |  |  |  |
| Paralarvae |  |  |  |  |  |  |  |
| 13ºC | Replicate 1 | 30,9 | 32,2 | 0,8099 | 8,5 | 0,100 | y = -1.658x + 32.195 |
|  | Replicate 2 | 31,4 | 33,3 | 0,9242 | 36,6 | 0,009 | y = -2.4308x + 33.321 |
|  | Replicate 3 | 31,8 | 33,2 | 0,9234 | 24,1 | 0,039 | y = -1.7855x + 33.197 |
|  |  |  |  |  |  |  |  |
| 15ºC | Replicate 1 | 31,4 | 33,3 | 0,8714 | 20,3 | 0,020 | y = -2.4169x + 33.346 |
|  | Replicate 2 | 31,6 | 33,2 | 0,8795 | 21,9 | 0,018 | y = -2.2508x + 33.179 |
|  | Replicate 3 | 31,3 | 32,7 | 0,9603 | 48,4 | 0,020 | y = -1.8553x + 32.742 |
|  |  |  |  |  |  |  |  |
| 17ºC | Replicate 1 | 32,1 | 33,1 | 0,9776 | 43,7 | 0,096 | y = -1.2448x + 33.063 |
|  | Replicate 2 | 32,1 | 33,1 | 0,9643 | 27,0 | 0,121 | y = -1.2278x + 33.071 |
|  | Replicate 3 | 32,1 | 33,1 | 0,9776 | 43,7 | 0,096 | y = -1.2448x + 33.063 |
|  |  |  |  |  |  |  |  |
| 19ºC | Replicate 1 | 34,2 | 35,1 | 0,8959 | 8,6 | 0,209 | y = -1.1407x + 35.072 |
|  | Replicate 2 | 33,2 | 35,0 | 0,9299 | 39,8 | 0,008 | y = -2.2326x + 34.97 |
|  | Replicate 3 | 33,6 | 35,1 | 0,9557 | 43,2 | 0,022 | y = -1.9432x + 35.126 |
