# Supplementary material for: Ocean Warming Enhances Malformations, Premature Hatching, Metabolic Suppression and Oxidative Stress in the Early Life Stages of a Keystone Squid
Source: PLoS One. 2012 Jun 6;7(6):e38282. doi: 10.1371/journal.pone.0038282 (PMC3368925; doi:10.1371/journal.pone.0038282)
Supplement: Table S4 — Pearson correlation coefficients between oxygen consumption rates (RMR) and thermal tolerance limits (LT50 and LT100) in the late embryos and paralarvae of Loligo vulgaris. (DOCX) [file pone.0038282.s004.docx]

**Supporting Information**

Table S4. Pearson correlation coefficients between oxygen consumption rates (RMR) and thermal tolerance limits (LT50 and LT100) in the late embryos and paralarvae of *Loligo vulgaris.*

|  |  | OCR | LT50 | LT100 |
| --- | --- | --- | --- | --- |
| Late embryos | OCR | 1.00 |  |  |
|  | LT50 | 0.94* | 1.00 |  |
|  | LT100 | 0.87 | 0.85 | 1.00 |
|  |  |  |  |  |
| Paralarvae | OCR | 1.00 |  |  |
|  | LT50 | 0.92* | 1.00 |  |
|  | LT100 | 0.86 | 0.98* | 1.00 |

* indicate statistical significance at the 5% level of significance.
