# Supplementary material for: Ocean Warming Enhances Malformations, Premature Hatching, Metabolic Suppression and Oxidative Stress in the Early Life Stages of a Keystone Squid
Source: PLoS One. 2012 Jun 6;7(6):e38282. doi: 10.1371/journal.pone.0038282 (PMC3368925; doi:10.1371/journal.pone.0038282)
Supplement: Table S5 — Pearson correlation coefficients between oxygen consumption rates (OCR), heat shock protein 70 (HSP70/HSC70), catalase (CAT), superoxide-dismutase (SOD), glutathione S-transferase GST and malondialdehyde (MDA) concentrations in the paralarvae of Loligo vulgaris. (DOCX) [file pone.0038282.s005.docx]

**Supporting Information**

Table S5. Pearson correlation coefficients between oxygen consumption rates (OCR), heat shock protein 70 (HSP70/HSC70), catalase (CAT), superoxide-dismutase (SOD), glutathione S-transferase GST and malondialdehyde (MDA) concentrations in the paralarvae of *Loligo vulgaris.*

|  |  | OCR | HSP70/HSC70 | GST | CAT | SOD | MDA |
| --- | --- | --- | --- | --- | --- | --- | --- |
| Late embryos | OCR | 1.00 |  |  |  |  |  |
|  | HSP70/HSC70 | 0.72 | 1.00 |  |  |  |  |
|  | GST | -0.32 | 0.37 | 1.00 |  |  |  |
|  | CAT | -0.48 | -0.09 | 0.23 | 1.00 |  |  |
|  | SOD | 0.26 | 0.44 | 0.54 | -0.69 | 1.00 |  |
|  | MDA | 0.94* | 0.77 | -0.07 | -0.65 | 0.58 | 1.00 |
|  |  |  |  |  |  |  |  |
| Paralarvae | OCR | 1.00 |  |  |  |  |  |
|  | HSP70/HSC70 | 0.96* | 1.00 |  |  |  |  |
|  | GST | 0.85* | 0.77 | 1.00 |  |  |  |
|  | CAT | 0.79 | 0.90* | 0.41 | 1.00 |  |  |
|  | SOD | 0.60 | 0.81* | 0.40 | 0.86* | 1.00 |  |
|  | MDA | 0.94* | 0.92* | 0.96* | 0.66 | 0.63 | 1.00 |

* indicate statistical significance at the 5% level of significance.
